# Arterial Stiffness in a Toddler with Neurofibromatosis Type 1 and Refractory Hypertension

**DOI:** 10.1155/2018/5957987

**Published:** 2018-10-31

**Authors:** Stella Stabouli, Euthymia Vargiami, Olga Maliachova, Nikoleta Printza, John Dotis, Maria Kyriazi, Konstantinos O. Papazoglou, Dimitrios Zafeiriou

**Affiliations:** ^1^1st Department of Pediatrics, Aristotle University Thessaloniki, Hippokration Hospital, Thessaloniki, Greece; ^2^5th Department of Surgery, Aristotle University of Thessaloniki, Hippokration Hospital, Thessaloniki, Greece

## Abstract

Arterial hypertension is a common finding in patients with neurofibromatosis (NF) type 1. Renovascular hypertension due to renal artery stenosis or midaortic syndrome could be the underlying cause. We report the case of a 4-year-old girl with NF type 1 and midaortic syndrome whose changes in blood pressure and pulse wave velocity suggested the evolution of vasculopathy, diagnosis of renovascular hypertension, and provided insights of response to treatment. Hypertension persisted after percutaneous transluminal angioplasty in the abdominal aorta, requiring escalation of antihypertensive treatment, while arterial stiffness demonstrated a mild decrease. Regular assessment of blood pressure using ambulatory blood pressure monitoring and noninvasive assessment of arterial stiffness may enhance the medical care of patients with NF type 1.

## 1. Introduction

Arterial hypertension is a common finding in patients with neurofibromatosis (NF) type 1 with a reported prevalence up to 20% [1–4]. Hypertension in NF type 1 is mainly secondary, either renovascular due to arterial dysplasia, or external compression of renal arteries by neurofibromas or other adjacent masses, while pheochromocytoma may be the underlying cause in older ages [1]. A broad range of vascular abnormalities can be seen in patients with neurofibromatosis type 1 including midaortic syndrome, which is characterized by severe narrowing of the abdominal aorta, usually involving the renal arteries and visceral branches [4]. Kaas et al. in a retrospective study including 181 pediatric patients showed that 18% of the children suffered from vascular abnormalities, with peripheral vasculopathy in 1% and cerebrovascular abnormalities in 7% [4].

Arterial stiffness is a general term used to describe the elasticity or compliance of the arteries [5]. The pathogenesis and etiology of the artery hardening is multifactorial, as structural, neuroendocrine, inflammatory, and genetic factors are involved. Arterial stiffness presence has been associated with increased cardiovascular risk, chronic renal disease, and arterial hypertension [6, 7]. Arterial stiffness can be assessed noninvasively through the determination of the carotid-femoral pulse wave velocity (cf-PWV), which is currently considered the gold standard method [5].

We report a case of refractory hypertension accompanied with increased arterial stiffness in a female toddler with NF type 1 and midaortic. We describe changes in office, ambulatory blood pressure (BP), and cf-PWV during follow-up visits suggesting evolution of acquired vasculopathy, diagnosis of renovascular hypertension, and provided insights of response to treatment.

## 2. Case Presentation

We report the case of a 4-year-old girl, who was diagnosed with NF type 1 at the age of 14 days. Her mother and her older brother also suffer from NF type 1. During the annual follow-up at the age of 2.5 years, she had office BP levels below the 90th percentile (p.c.) for age, sex, and height (105/59 mmHg, 98 bpm). A routine ambulatory blood pressure monitoring (ABPM) showed a nondipping profile with normal mean BP levels (mean 24 h BP 107/61 mmHg, mean 24 h HR 99 bpm) (Table 1).

**Figure 1:**
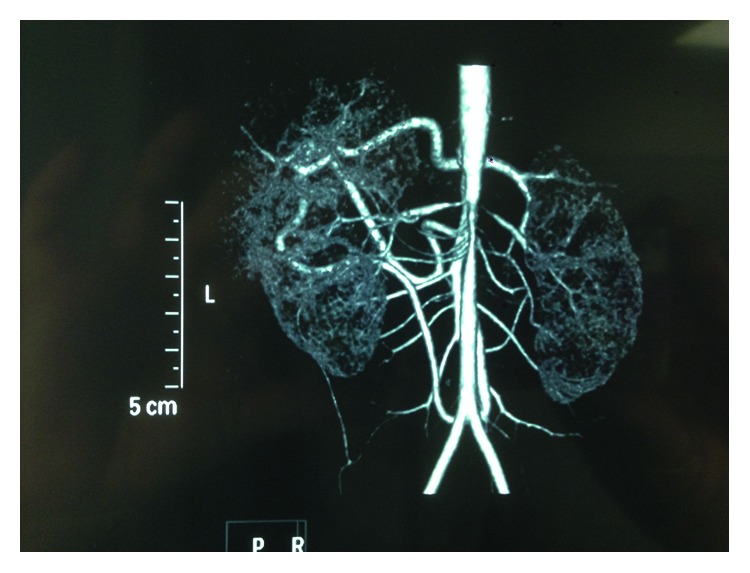
CTA at diagnosis showed severe segmental aortic stenosis arising before the origin of the upper mesenteric artery extending for a length of 3–4 cm with lumen diameter of 0.2–0.3 cm at the narrowest part and a poststenotic diameter of 0.6–0.7 cm. Two right renal arteries were demonstrated, both with ostial stenosis, as well as an auxiliary stenotic artery perfusing the lower pole of the right kidney. Left renal artery also presented ostial stenosis. Furthermore, an enlarged riolan arcade resulted in an increased blood flow to the upper mesenteric artery from the lower mesenteric artery providing collateral circulation.

One year later, at the age of 4 years, her office BP was greater than the 95th p.c., and a difference of 20 mm Hg between upper and lower extremities was documented (Table 1). Clinical examination also showed a systolic murmur of 3/6 with punctum maximum on the Erb point, weak femoral and pedal pulses, and absent tibial posterior pulses on both sides. Other clinical findings were multiple cafe au lait signs on her whole body and underdevelopment of the left leg. ABPM revealed daytime and nighttime hypertension (Table 1). The patient underwent the necessary laboratory and imaging examinations to diagnose the cause of hypertension, and she commenced on valsartan with the addition of felodipine because of inadequate BP control (Table 1). Laboratory exams showed normal renal function. Renal ultrasound demonstrated a right kidney length of 7.1 cm and a left kidney length of 8.5 cm. Echocardiography did not reveal left ventricular hypertrophy or any evidence of other cardiac anatomical or functional abnormalities. Fundoscopy was normal. Measurement of cf-PWV (SpygmoCor, AtCor Medical) showed increased arterial stiffness (Table 1). Magnetic resonance imaging (MRI) and magnetic resonance angiography (MRA) imaging of the brain, spinal column, and abdomen and X-ray examination of all long bones revealed multiple brain hamartomas and one neurinoma at the 10th vertebra of the thoracic spine. Magnetic resonance angiography of the aorta and renal arteries showed abdominal aortic stenosis (lumen diameter 0.30 cm) and inability of imaging the arise of right renal artery. A subsequent computed tomography angiography (CTA) documented severe segmental aortic stenosis arising before the origin of the upper mesenteric artery and including the origin of renal arteries. Renal arteries also presented ostial stenosis (Figure 1). The patient underwent percutaneous transluminal angioplasty (PTA) in the abdominal aorta, while direct dilatation of renal arteries was not possible to perform due to small diameter of renal arteries. There was deterioration in renal function after angioplasty due to contrast-induced acute renal injury accompanied by deterioration of BP levels. A DTPA (99mTc-diethylenetriaminepentaacetic acid) renal scan showed 25.8% left kidney contribution in the total renal function.

On her follow-up visit 4 months after PTA, there was no difference in BP between the upper and lower extremities, and pulses were present on both sides. Office BP levels presented a decrease but remained greater than the 95th percentile. Moreover, 24 h BP increased and cf-PWV was further increased, despite pharmacological treatment with amlodipine, furosemide, and atenolol. Clonidine was subsequently added to treatment. On the other hand, renal function was significantly improved. Both MRA and CTA showed a moderate improvement of the anatomical structures with abdominal aorta stenosis ranging between 0.35 and 0.5 cm, while other findings remained constant.

During her next follow-up visit, 8 months after PTA, renal function was further improved, and the patient presented significant reduction of ambulatory BP levels and a reduction in cf-PWV values despite sustained office BP elevation (Table 1). However, at 12 months after angioplasty, ambulatory BP levels were again increased at preangioplasty levels. In contrast, cf-PWV was further decreased, while MRA findings of the aorta and renal arteries remained unchangeable.

## 3. Discussion

We present a case of refractory hypertension in a toddler with NF type 1 and midaortic syndrome. The diagnosis of hypertension due to midaortic syndrome in our patient was an incidental finding. Severe symptoms associated with BP elevation have been reported in cases series of children with midaortic syndrome [8]. The early diagnosis of hypertension and probably the short duration of BP elevation in the patient may explain the absence of clinical symptoms. However, subclinical damage as demonstrated by increase in arterial stiffness was already present. Moreover, hypertension persisted after PTA requiring further escalation on antihypertensive medication.

Neurofibromatosis type 1 has been reported as being the most common genetic condition associated with midaortic syndrome [6]. Tummolo et al. reported that severe hypertension in children with midaortic syndrome is usually refractory to pharmacological treatment requiring PTA and/or surgical treatment [8]. PTA may not result in HTN control in a significant proportion of patients with renovascular hypertension. Hypertensive children having complex, more widespread stenosis may present the poorest outcome after angioplasty, while clinical benefit has been reported in 79.9 % of the children with involvement of only the main renal arteries [9]. Reconstructive surgery would provide better outcomes, but is preferably delayed until school age or adolescence as younger children may present smaller postoperative benefit and need for high number of reoperations [10].

The correlation between arterial BP and arterial stiffness has already been well documented in previous pediatric studies [6, 7]. In the Bogalusa Heart Study, it was shown that systolic BP is a predictor of arterial stiffness in healthy asymptomatic young individuals [11]. On the other hand, Tedesco et al. found no difference in cf-PWV in children with NF type 1 compared with healthy controls [12]. The presence of severe hypertension could explain the presence of increased arterial stiffness in our patient. Office BP levels in the patient presented a decrease at 4 months after PTA. In contrast, ambulatory BP levels increased at 4 months after angioplasty and were accompanied by an increase in cf-PWV. Increased BP variability may explain the presence of this discrepancy [6]. The superiority of ABPM in the assessment of hypertension in children and its close relation to target organ damage has been previously described [13]. In our case, ambulatory BP levels seem to better reflect cf-PWV values. The continuing reduction in cf-PWV levels despite the high ABP in the last follow-up visit could be attributed to the direct effect of antihypertensive medication on arterial stiffness [14]. Dihydropyridine calcium channel blockers have been shown to have antioxidant effects in animal models [15]. Moreover, adults in randomized control studies showed a reduction in cf-PWV in patients treated with amlodipine compared to placebo. Clinical benefit of antihypertensive treatment on arterial stiffness independently of BP response may have important prognostic implications.

Regular BP assessment enables early diagnosis of hypertension in genetic conditions with high prevalence of midaortic syndrome, which recent studies show to present in younger ages than initially reported [1, 8]. Furthermore, early recognition of BP abnormalities may be crucial in order to diagnose early evolution of arterial stenosis in the context of acquired neurofibromatosis vasculopathy. The presence of nondipping profile at the age of 3 years may reflect the development of new vascular lesions, which progressed to clinical presentation of hypertension one year later. Tedesco et al. highlighted the value of ABPM in the assessment of NF type 1 pediatric patients showing that masked hypertension may be present in these patients and that nondipping profile was common among those with ambulatory hypertension [12]. On the other hand, dipping status has been reported to present a low reproducibility in pediatric patients, and current guidelines are not supporting investigation upon this [13, 16].

In conclusion, renovascular hypertension in NF type 1 may have significant adverse effect on arterial stiffness event with short duration of HTN. Regular assessment of BP using ABPM may enhance the medical care of patients with NF type 1 during regular assessment for the diagnosis of cardiovascular complications and guide further management. The use of more noninvasive techniques including cf-PWV could further add on the prognosis and evaluation of therapeutic outcome in patients with refractory hypertension.

## Figures and Tables

**Table 1:** Clinical, laboratory, and imaging data during follow-up visits.

	Age (yrs)	Height (cm)	Office BP (mmHg)/BP pc	Ambulatory BP (mean day/night, mmHg)	Office central BP^1^ (mmHg)	Cf-PWV (m/s)^2^	MRA aortic diameter (cm)	CTA aortic diameter (cm)	DTPA scan (right) % (left) %	Renin active/rest (ng/ml/min) aldosterone active/rest (pg/dl)^3^	Creatinine (mg/dl)/eGFR (ml/min/1.73 m^2^)^4^	Urine protein (mg/m^2^/24h)	Medication^5^
18 months	2.5	95	101/60 <90th	107/61									
				106/61									
				108/61									
1 month	4	98	125/85 >99th	122/80	116/86	5	0.3	0.2–0.3		8.75/30.94	0.58/93	41.2	
				123/81									
				119/77									

Before↑ After↓	**PTA**												
1 month	4	99	131/80 >99th				0.35–0.5		25.8/74.2		0.79/68		Valsartan felodipine
4 months	4	100	118/73 95–99th	127/76	111/77	5.6		0.34–0.38	17.7/82.3		0.72/76	61.5	Amlodipine
				128/77									Furosemide
				125/73									Atenolol
8 months	5	103	133/91 >99th	112/65	127/93	5				4.08/1.68	0.73/77	49.4	Amlodipine
				115/67									Furosemide
				106/56						10.8/8.52			Atenolol
													Clonidine
12 months	5	104	135/81 >99th	128/76	127/86	4.6	0.35		27/73	5.84/1.06	0.73/78	48	Amlodipine
				122/77						12.3/12.4			Furosemide atenolol
				115/74									Clonidine

^1^Central BP was measured by an oscillometric device (SpygmoCor, AtCor Medical). ^2^Reference values available for children older than 7 yrs.: 5.26 m/sec 95th percentile for 7-year-old girls, 5.06 m/sec 95th percentile for 7-year-old girls. ^3^eGFR was based on Schwartz formula. ^4^Renin and aldosterone at rest was measured after night's rest in the supine position and then active after 1 hour walking in the upright position.^5^ Antihypertensive drugs doses: valsartan:1.3 mg/kg/day qd; felodipine: 2.5 mg daily qd; amlodipine: 0.5 mg/kg/day qd; furosemide: 1 mg/day bid; atenolol:1 mg/kg/day bid; clonidine: 0.1 mg/day bid. BP: blood pressure; cf-PWV: carotid-femoral pulse wave velocity; MRA: magnetic resonance angiography; CTA: computed tomography angiography; DTPA: 99mTc-diethylenetriaminepentaacetic acid.
